# Multiresponsive Cellulose Nanocrystal Cross-Linked Copolymer Hydrogels for the Controlled Release of Dyes and Drugs

**DOI:** 10.3390/polym13081219

**Published:** 2021-04-09

**Authors:** Yuchen Jiang, Guihua Li, Chenyu Yang, Fangong Kong, Zaiwu Yuan

**Affiliations:** 1 Key Laboratory of Fine Chemicals in Universities of Shandong, School of Chemistry and Chemical Engineering, Qilu University of Technology (Shandong Academy of Sciences), Jinan 250353, China; 1043117140@stu.qlu.edu.cn (Y.J.); 1043118122@stu.qlu.edu.cn (C.Y.); 2 State Key Laboratory of Biobased Material and Green Papermaking, Qilu University of Technology (Shandong Academy of Sciences), Jinan 250353, China; kfg@qlu.edu.cn

**Keywords:** hydrogels, cellulose nanocrystals, UCST polymer, hydrogen bond, drug release

## Abstract

Multiresponsive hydrogels have attracted tremendous interest due to their promising applications in tissue engineering, wearable devices, and flexible electronics. In this work, we report a multiresponsive upper critical solution temperature (UCST) composite hydrogel based on poly (acrylic acid-co-acrylamide), PAAc-co-PAAm, sequentially cross-linked by acid-hydrolysis cellulose nanocrystals (CNCs). Scanning electron microscopy (SEM) observations demonstrated that the hydrogels are formed by densely cross-linked porous structures. The PAAc/PAAm/CNC hybrid hydrogels exhibit swelling and shrinking properties that can be induced by multiple stimuli, including temperature, pH, and salt concentration. The driving force of the volume transition is the formation and dissociation of hydrogen bonds in the hydrogels. A certain content of CNCs can greatly enhance the shrinkage capability and mechanical strength of the hybrid hydrogels, but an excess addition may impair the contractility of the hydrogel. Furthermore, the hydrogels can be used as a matrix to adsorb dyes, such as methylene blue (MB), for water purification. MB may be partly discharged from hydrogels by saline solutions, especially by those with high ionic strength. Notably, through temperature-controlled hydrogel swelling and shrinking, doxorubicin hydrochloride (DOX-HCl) can be controllably adsorbed and released from the prepared hydrogels.

## 1. Introduction

Polymer hydrogels are three-dimensional polymer networks that can hold a large amount of water in the interspaces of their network [1,2,3,4,5]. Smart hydrogels are stimulus-responsive materials that can intelligently respond to environmental stimuli, such as the temperature and pH, along with the presence of electricity, a magnetic field, and light [6,7,8,9,10]. During the response process, smart hydrogels exhibit drastic volume changes and volume phase transitions; thus, they can be widely applied in wastewater treatment, drug delivery systems, and soft robotics [11,12,13]. Numerous polymers have been used to synthesize multiresponsive hydrogels, such as poly(N-isopropyl acrylamide) (PNIPAM) [14], polyacrylamide (PAAm) [15], poly (acrylic acid) (PAAc) [16], and poly(N, N-diethylacrylamide) [17]. It is well established that the volume change of hydrogels can be induced by individual or a combination of molecular interactions, including ionic interactions, hydrophobic interactions, hydrogen bonding, and van der Waals forces.

Lower critical solution temperature (LCST) and upper critical solution temperature (UCST) are two major categories of thermal transitions for thermoresponsive polymers. Poly(2-(dimethylamino)-ethyl methacrylate) (PDMAEMA) and PNIPAM are typical LCST-type polymers and have been widely reported [18]. Notably, most thermoresponsive hydrogels are based on polymers with LCSTs, and polymers with UCSTs are rarely reported in the fabrication of smart hydrogels [19,20,21]. Some researchers have found that nonresponsive polymers can be converted into thermoresponsive polymers in water by controlling the strength of polymer–polymer interactions. For example, thermoresponsive hydrogels were constructed with the incorporation of PAAm, which is not a thermoresponsive polymer. When polymer–polymer hydrogen bonds are stronger than polymer–water bonds, UCST polymers in water can be obtained. Dai and coworkers designed smart hydrogels with UCST characteristics by constructing an interpenetrating network (IPN) of PAAm and PAAc with chlorophyllin incorporated as the chromophore [16].

Cellulose is one of the most abundant natural biopolymers, composed of glucose, and is responsible for the structural scaffolding of cells in all green plants [22]. Because of their intrinsic biocompatibility and biodegradability, cellulose-based functional materials have attracted great attention in scientific research. Needle-like cellulose nanocrystals (CNCs), derived from acid hydrolysis, have been widely used to reinforce nanocomposite hydrogels [23]. Chang et al. reported a nanocomposite network of poly(acrylic acid-co-acrylamide) (PAAAM) sequentially cross-linked by quaternized tunicate cellulose nanocrystals (Q-TCNCs) and Fe^3+^ [24]. Q-TCNCs contain many hydroxyl groups, the surfaces of which are positively charged. In the nanocomposite hydrogels, Q-TCNCs act as both interfacial compatible reinforcers and cross-linkers. Nanocomposite hydrogels reinforced by poly(N-vinylpyrrolidone)-grafted cellulose nanocrystals (CNCs-g-PVP) were developed by a dual physical cross-linking strategy [25]. The obtained hydrogels exhibited high tensile strength, remarkable toughness, rapid self-recovery, and favorable fatigue resistance. The introduction of CNCs into polymer networks not only enriches raw material choices for finite components but also facilitates the formation of multiple hydrogen bonds, the formation and dissociation of which are important for understanding the swelling and shrinking properties of polymer hydrogels.

**Scheme 1:**
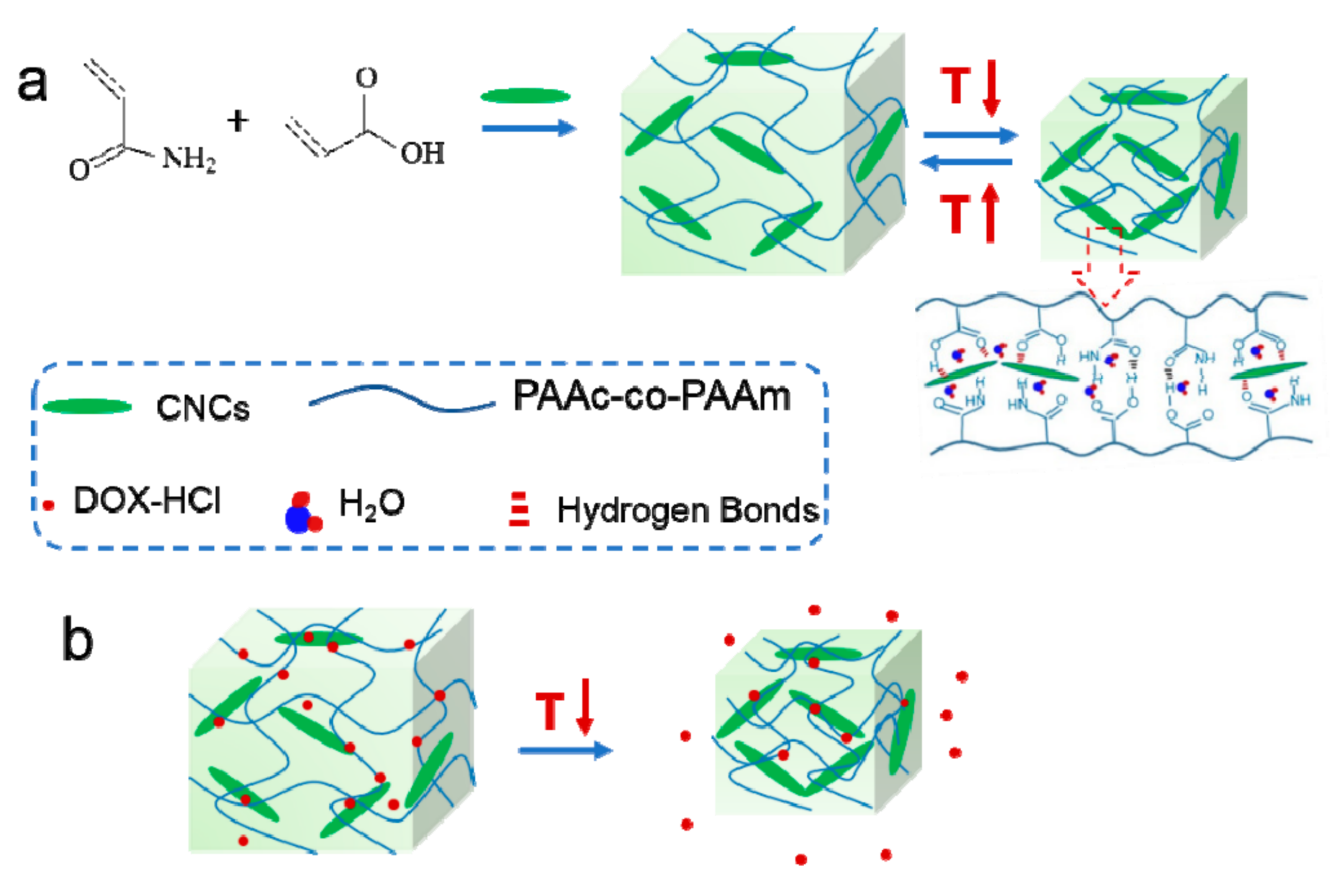
(**a**) Synthesis route of PAAc/PAAm/CNC hydrogels; the shrinkage property induced by the temperature is mainly derived from the formation of hydrogen bonds between the polymer chains and CNCs; (**b**) Schematic illustration of the release of doxorubicin hydrochloride (DOX-HCl) from the hydrogel.

Herein, a series of CNC-reinforced nanocomposite hydrogels were fabricated by incorporating CNCs into the polymer network of PAAc-co-PAAm through hydrogen bonds, denoted as PAAc/PAAm/CNC (Scheme 1). The carboxylic acid (–COOH) groups of PAAc and amide (–CONH_2_) groups of PAAm form intra- and intermolecular hydrogen bonds only at low temperature, while they dissociate at a high temperature. The so-called “zipper effect” also exists in the PAAc/PAAm/CNC hydrogels. Deprotonation of the carboxy groups of PAAc and the amide groups of PAAm at high pH weakens the hydrogen bonds, which induces swelling of the prepared hydrogels. According to the “Hofmeister series” effect, the sulfate ester (–SO_3_H) groups on the CNC surface help to precipitate polymers, which drives hydrogels to shrink. The cross-sectional scanning electron microscopy (SEM) results show that interconnected porous structures were formed in the PAAc/PAAm/CNC hydrogels. The shrinkage capability and toughness of the PAAc/PAAm/CNC hydrogels are significantly improved compared to those of the PAAm-co-PAAc hydrogel. Based on these properties, the prepared hydrogels were used as a matrix to efficiently adsorb toxic dyes. Additionally, through hydrogel swelling and shrinking, doxorubicin hydrochloride (DOX-HCl) that is loaded in the hydrogels can be reversibly and controllably released and adsorbed in solution.

## 2. Materials and Methods 

### 2.1. Materials

The monomers acrylamide (AAm, 98%) and acrylic acid (AAc, AR grade) and the initiator potassium persulfate (KPS, AR grade) were obtained from Sinopharm Chemical Reagent Co., Ltd. (Shanghai, China), H_2_SO_4_, HCl, NaOH, MgCl_2_, and NaCl were purchased from Tianjin Damao Chemical Reagent Factory (Tianjin, China), N,N’-Methylenebis(acrylamide) (MBA) was supplied by Shanghai Mackin Biochemical Co., Ltd. (Shanghai, China), doxorubicin hydrochloride (DOX-HCl) was purchased from Dalian MeiLun Biotechnology Co., Ltd. (Dalian, China), and methylene blue (MB) and methyl orange (MO) were purchased from Wenzhou Red Flag Auxiliary Factory (Wenzhou, China). Milli-Q water was used in all experiments.

### 2.2. Preparation of the Cellulose Nanocrystals (CNCs)

Milled cellulose pulp was hydrolyzed by 64 wt% H_2_SO_4_ (8.5 mL solution/1 g pulp) at 45 °C and stirred for 30 min. Then, the reaction was terminated by adding 1000 mL of cold water. After undergoing sedimentation overnight, the bottom CNC suspension was removed, then alternately centrifuged and washed 3 times with ultrapure water. The obtained concentrated CNC suspension was placed inside dialysis membrane bags (with an 8000–14,000 molecular weight cutoff) for dialysis for ~7 days until the pH reached 6. The dialyzed suspension was sonicated for 10 min at 90 W and then evaporated to obtain a 3 wt% CNC dispersion. For the desulfation of CNCs, a 3 wt% CNC dispersion (270 mL) was mixed with NaOH (10.8 g), and the mixture was stirred at 60 °C for 5 h. The obtained suspension was purified by dialysis against Milli-Q water until the effluent remained at neutral pH, resulting in a desulfated CNC (DCNC) suspension.

### 2.3. Desulfation Procedure of the Cellulose Nanocrystals (CNCs) 

The sulfate ester groups were removed as previously described by Grey et al. [26]. A 3.68% *w/w* CNC suspension (250 mL) was mixed with NaOH (10 g) to give a concentration of 1 M NaOH, and the mixture was stirred at 60 °C. The mixture turned slightly yellowish, and the nanoparticles started to aggregate and settle. After 5 h the reaction was stopped, and the suspension was purified by dialysis against deionized water until the effluent remained at neutral pH. The weight percentage of the resulting CNC suspension was 2.73%, and the yield of the reaction was 75%. The sulphur contents of the CNCs before and after desulfation were measured to be 0.81 ± 0.04 wt% and 0.27 ± 0.03 wt%, respectively, by a conductometric titration method [27].

### 2.4. Preparation of the PAAc/PAAm/CNC Composite Hydrogels

Quantities of 0.13 g of AAc, 0.13 g of AAm (*n*_AAc_/*n*_AAm_ molar ratio of approximately unity), 0.0075 g of KPS, and 0.0065 g of MBA (2.5 wt% of the total weight of the monomers) were added to 5 g of dispersions respectively containing 1 wt%, 2 wt%, or 3 wt% CNCs. These solutions were heated at 75 °C for 6 h, resulting in the PAAc/PAAm/CNC composite hydrogels, which were denoted as PAAc/PAAm/CNC_x_ (x = 1, 2, or 3, representing the 1 wt%, 2 wt%, or 3 wt% CNC dispersion, respectively).

### 2.5. Shrinking/Swelling Measurement of the Hydrogels

The shrinking/swelling properties of the PAAc/PAAm/CNC hydrogels at various temperatures and pH values and in various saline solutions were evaluated by weighing them after keeping them at equilibrium for an adequate amount of time. For example, to observe the swelling response to varying pH, the hydrogels were cut into pieces of the same weight (~0.5 g) and then immersed in 10 mL of solutions at various pH values (such as 2, 6, and 12) and kept at equilibrium for 24 h before measurement. For examination of the response to alternating temperatures between 2 °C and 37 °C, the pH was fixed at 2 or 4. In addition, two representative salt solutions, NaCl and MgCl_2_, were used to detect the swelling–shrinking response to salt solutions at various ionic strengths.

### 2.6. Dye/Drug Loading and Release

First, the maximum uptakes of MB and DOX-HCl in the PAAc/PAAm/CNC_2_ hydrogel were determined. A PAAc/PAAm/CNC_2_ hydrogel of 0.50 g was immersed into a 10 mL solution of 0.1 mg·mL^−1^ MB for 3 days at 25 °C for adequate adsorption. The maximum uptake was measured to be 16.3 milligrams of MB per gram of hydrogel, and the resulting hydrogel was denoted MB@PAAc/PAAm/CNC_2_. Under the same conditions, a 0.53 g PAAc/PAAm/CNC_2_ hydrogel was immersed in 10 mL aqueous solution of 0.85 mg·mL^−1^ DOX-HCl, resulting in a DOX-HCl@PAAc/PAAm/CNC_2_ hydrogel with a maximum uptake of 13.3 milligrams of DOX-HCl per gram of hydrogel. Thereafter, release experiments were carried out by immersing the above as-prepared MB@PAAc/PAAm/CNC_2_ and DOX-HCl@PAAc/PAAm/CNC_2_ hydrogels in 10 mL of an aqueous phase at a fixed temperature and pH. The concentrations of MB and DOX-HCl in the media were monitored by taking 500 μL aliquots at specific time points. Every time, the media needed to be restored to 10 mL by water at the same pH value. The release or resorption concentrations in the media were determined at 664 nm (MB) and 484 nm (DOX-HCl) using a UV spectrophotometer. Every concentration point was repeated three times. Calibration curves of MB and DOX-HCl were made in advance using standard solutions at known concentrations.

### 2.7. Characterizations

Fourier transform infrared (FT-IR) spectroscopy was carried out on an FT-IR spectrometer (Thermo Fisher Scientific, 5225 Verona Rd, Fitchburg, WI, USA) in the wavenumber range of 4000–400 cm^−1^. X-ray diffraction (XRD) patterns for the dried hydrogels were obtained using a D8-ADVANCE diffractometer (Bruker, Germany) equipped with a Cu Kα X-ray source (λ = 0.15418 nm) and a graphite monochromator. SEM images were recorded on a field-emission microscope (Regulus 8220) at an accelerating voltage of 5 kV. The hydrogel specimens needed to be freeze-dried and fractured for SEM observation of the fractured surface. Thermal gravimetric analysis (TGA) was performed from 50 °C to 950 °C using a thermogravimetric analyzer (TGA Q50, Micromeritics Instrument Corporation) at 10 °C/min in nitrogen. Differential scanning calorimetry (DSC) experiments were carried out using a Q2000 DSC (TA instrument, New Castle, DE, USA) at 10 °C/min in nitrogen. Note that the samples for the FT-IR and XRD experiments were freeze-dried under vacuum before use. For DSC characterization, hydrogels were used without any treatment. The rheology of the PAAc/PAAm/CNC_x_ hydrogels was systematically measured on a HAAKE Rheostress 6000 with a Thermo Scientific heating system at two temperatures (4 °C and 25 °C), with all the specimens thermally equilibrated for 5 min before measurement. Hydrogel films with a 20 mm diameter were cut into 0.7 mm thick pieces and placed into a parallel plate geometry 45.0 mm in diameter. To determine the linear viscoelastic region of each hydrogel sample, the stress sweep was checked at a fixed frequency (1.0 Hz). In the drug loading, release, and resorption experiments, an ultraviolet–visible (UV–vis) spectrometer (UV-2600) was used to determine the UV–vis absorption spectra of the MB and DOX-HCl aqueous solutions.

## 3. Results and Discussion

### 3.1. Preparation and Characterization of the PAAc/PAAm/CNC Hydrogels

**Figure 1:**
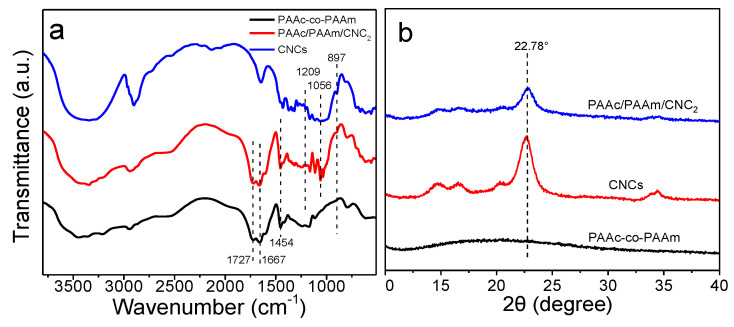
(**a**) FTIR spectra and (**b**) XRD patterns of CNCs and the PAAc-co-PAAm and PAAc/PAAm/CNC_2_ hydrogels.

A series of PAAc/PAAm/CNC hydrogels were synthesized by thermal polymerization at 75 °C. Photographs of typical PAAc/PAAm/CNC hydrogels kept at a high temperature (52 °C) are shown in Figure S1. Unlike the transparent PAAc-co-PAAm hydrogel, the PAAc/PAAm/CNC hydrogels exhibited an opaque appearance, and their transparency decreased with increasing CNC concentration. The FT-IR spectra of the freeze-dried as-prepared hydrogels (Figure 1a) were obtained to investigate the combination and hydrogen bonds between components. For pure CNCs (blue line), the surface of which has abundant –OH groups, the –OH stretching peak appeared at approximately 3650–3200 cm^−1^ [28]. A broad band from 3455 to 3100 cm^−1^ was observed for the PAAc-co-PAAm (black line) and PAAc/PAAm/CNC_2_ (red line) hydrogels, which is characteristic of hydrogen bonds between the −COOH groups of PAAc, –CONH_2_ groups of PAAm, and −OH groups of CNCs [28,29]. Two peaks appeared at 897 cm^−1^ and 1056 cm^−1^ for both the CNCs and the PAAc/PAAm/CNC_2_ hydrogel, which are characteristic of the asymmetrical stretching vibration peak of C–O–C and the bending vibration peak of C–O at the β-(1–4)-glycosidic linkage. In addition, the peak at 1209 cm^−1^ is attributed to the stretching vibration of S=O in the –OSO_3_H groups, which also exists in both CNCs and the PAAc/PAAm/CNC_2_ hydrogels. The stretching vibration of carbonyl at 1667 cm^−1^ and 1727 cm^−1^ and the deformation vibration of –NH_2_ at 1612 cm^−1^ were weakened for the PAAc/PAAm/CNC_2_ hydrogel, further indicating the formation of hydrogen bonds [30,31,32].

To detect the structural changes between pure CNCs and the hybrid hydrogels, the XRD patterns are shown in Figure 1b. For pure CNCs, typical cellulose I crystalline peaks at 14.9°, 16.4°, 22.8°, and 34.4° were observed [33,34,35]. Compared to the PAAc-co-PAAm hydrogel, the PAAc/PAAm/CNC_2_ hydrogel exhibited the characteristic diffraction peak of CNCs at 22.8°, although the strength of the peak was slightly lower. At the same time, the PAAc/PAAm/CNC_2_ hydrogel displayed a stronger amorphous nature than pure CNCs, as evidenced by its elevated broad peak. The diffraction peaks of the PAAc/PAAm/CNC_2_ hydrogels were assumed to be the superimposition of those of CNCs and the PAAc-co-PAAm hydrogel.

**Figure 2:**
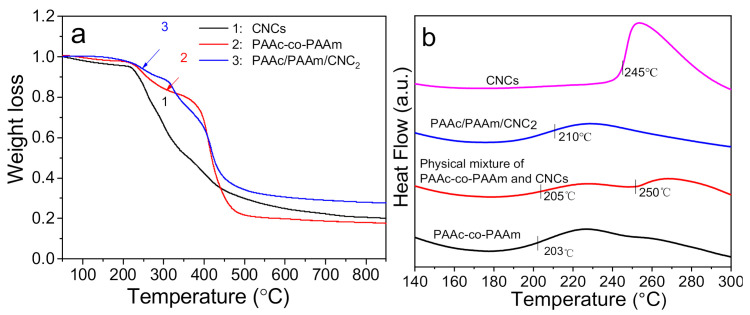
(**a**) TGA curves and (**b**) DSC thermograms of CNCs and the PAAc-co-PAAm and PAAc/PAAm/CNC_2_ hydrogels. For comparison, the DSC thermogram of the physical mixture of PAAc-co-PAAm and CNCs is shown.

The thermal properties of the PAAc/PAAm/CNC_2_ hydrogels were examined by thermogravimetric analysis (TGA) and differential scanning calorimetry (DSC). From the TGA curves shown in Figure 2a, less weight loss was recorded for the PAAc/PAAm/CNC_2_ hydrogel than for the hydrogel without CNCs in the range of 500–800 °C. The presence of CNCs helps to improve the thermal stability of the hybrid PAAc/PAAm/CNC hydrogels. The DSC curve is a useful tool to assess the miscibility and combination in polymer blends [16,36]. The single CNC component showed a prominent endothermic peak at 245 °C (Figure 2b and Figure S2), which may be attributed to the decomposition temperature of CNCs according to the TGA data. The individual dried PAAc-co-PAAm hydrogel showed one single glass transition temperature at 203 °C. In the case of the PAAc/PAAm/CNC_2_ hydrogel, it can be observed that the curve exhibits a single glass transition temperature located at 210 °C, which is approximately between those of the two individual components of CNCs and the PAAc-co-PAAm polymer. Dai et al. [16] reported that the glass transition *Tg* value of the PAAm–PAAc IPN was 125 °C. This suggests that the hydrogels formed in this work exhibited higher thermal stability than did the PAAm–PAAc hydrogels. Comparatively, for the physical mixture of CNCs and the PAAc-co-PAAm hydrogel (with a dry weight ratio of 1:1), two separate glass transition temperatures occurred. These results demonstrate the good combination and interaction between PAAc-co-PAAm and CNCs.

### 3.2. Shrinkage and Swelling Properties of the PAAc/PAAm/CNC Hydrogels

Due to temperature-dependent intermolecular interactions between PAAc and PAAm, the composite hydrogels composed of PAAc and PAAm exhibited remarkable shrinking–swelling properties with varying temperature. Specifically, at high temperatures, the PAAc and PAAm molecules in the hydrogels maintain a high dissociation state, while at a relatively low temperature, zipper-like cooperative hydrogen bonds can form between PAAc and PAAm, and water molecules are expelled from the hydrogel matrix [15,37,38]. Therefore, the IPN hydrogel composed of PAAc and PAAm possesses outstanding shrinking–swelling properties that are dependent on temperature. However, despite consisting of the same PAAc and PAAm segments, their copolymer, i.e., the PAAc-co-PAAm hydrogels, exhibited comparatively weak shrinking–swelling capability in response to various temperatures. Thus, the introduction of a small amount of CNCs to PAAc-co-PAAm can greatly improve the shrinking–swelling capability of the hydrogel.

#### 3.2.1. Temperature Effects

**Figure 3:**
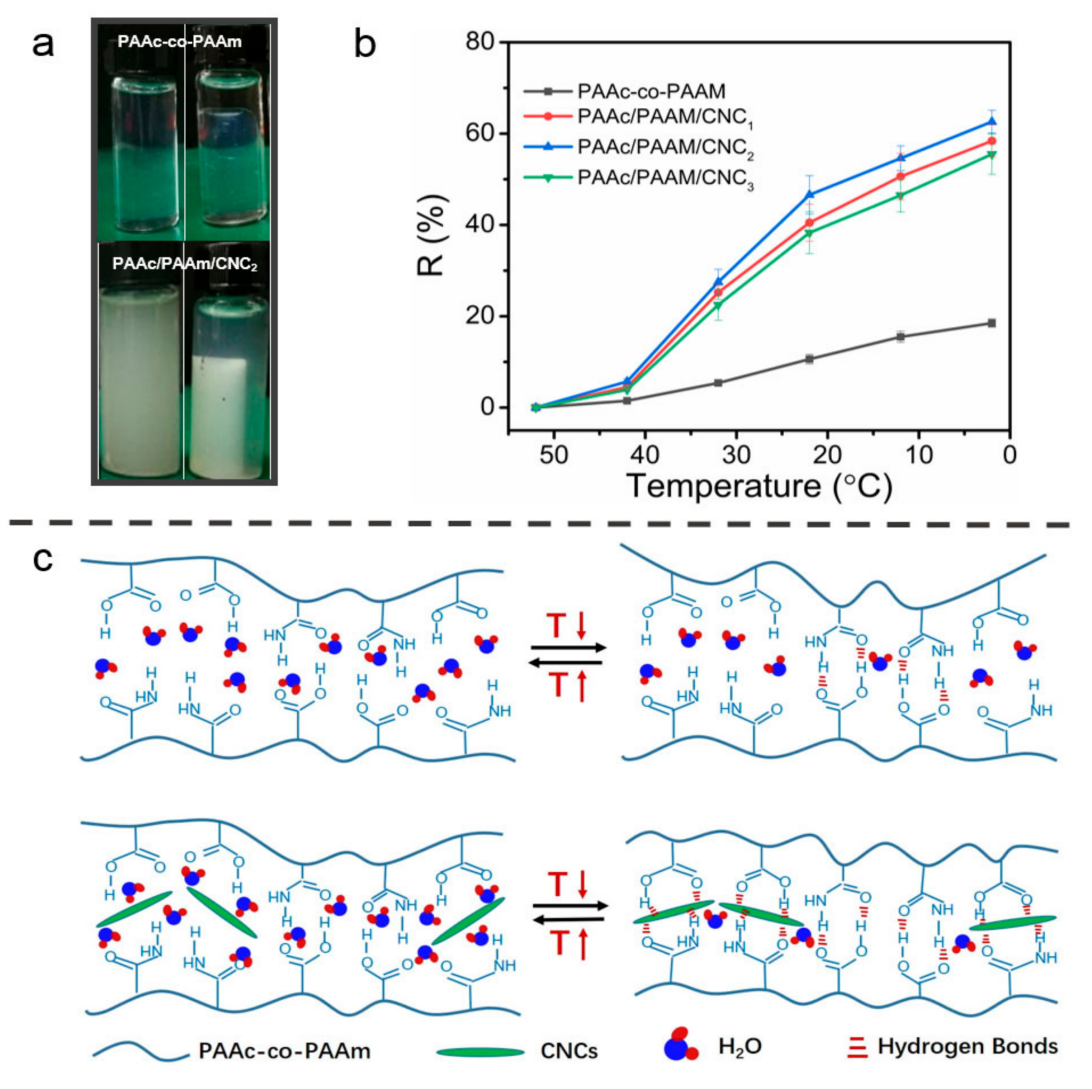
(**a**) Photographs of the PAAc-co-PAAm and PAAc/PAAm/CNC_2_ hydrogels at 52 °C (**left**) and 2 °C (**right**); (**b**) shrinkage ratio curves of the typical hydrogels at varying CNC concentrations and different temperatures; and (**c**) structural representations of both the copolymer hydrogels without and with CNCs, indicating their structural changes after temperature variation.

Similar to the PAAc–PAAm IPN hydrogels [8,39], the PAAc-co-PAAm hydrogels have an upper critical solution temperature (UCST), below which the hydrogel will shrink from a swollen volume. When the temperature was reduced from the original preparation temperature (75 °C) to 2 °C, the PAAc-co-PAAm hydrogel (*n*_AAc_/*n*_AAm_ = 1:1) showed a volume shrinkage accompanied by water expulsion from the hydrogel matrix (Figure 3a). The shrinkage ratio, *R*, is calculated via Equation (1):

*R* = *(m*_52_ − *m*_t_)/*m*_52_ (1)

where *m*_52_ is the weight of the hydrogel at 52 °C and *m*_t_ is that at *t* °C. It should be noted that the weight and volume of the hydrogels at 52 °C remained the same as those at 75 °C. From Figure 3b, the shrinkage of the PAAc-co-PAAm hydrogel was significantly low, merely 18.5%. It is well known that in the PAAc-co-PAAm hydrogels, the network is interwoven by the direct covalent linkages of polymer chains. These linkages enhance the rigidity of the hydrogel network to a certain extent but may simultaneously prevent cross-linked polymer chains from migrating and further associating through H-bonds (Figure 3c). Thus, only a small number of H-bonds can form between the PAAc and PAAm segments when the temperature is reduced. This leads to the PAAc-co-PAAm hydrogels having a relatively weaker shrinkage capability than the PAAc-PAAm IPN hydrogels [16,40]. 

**Scheme 2:**
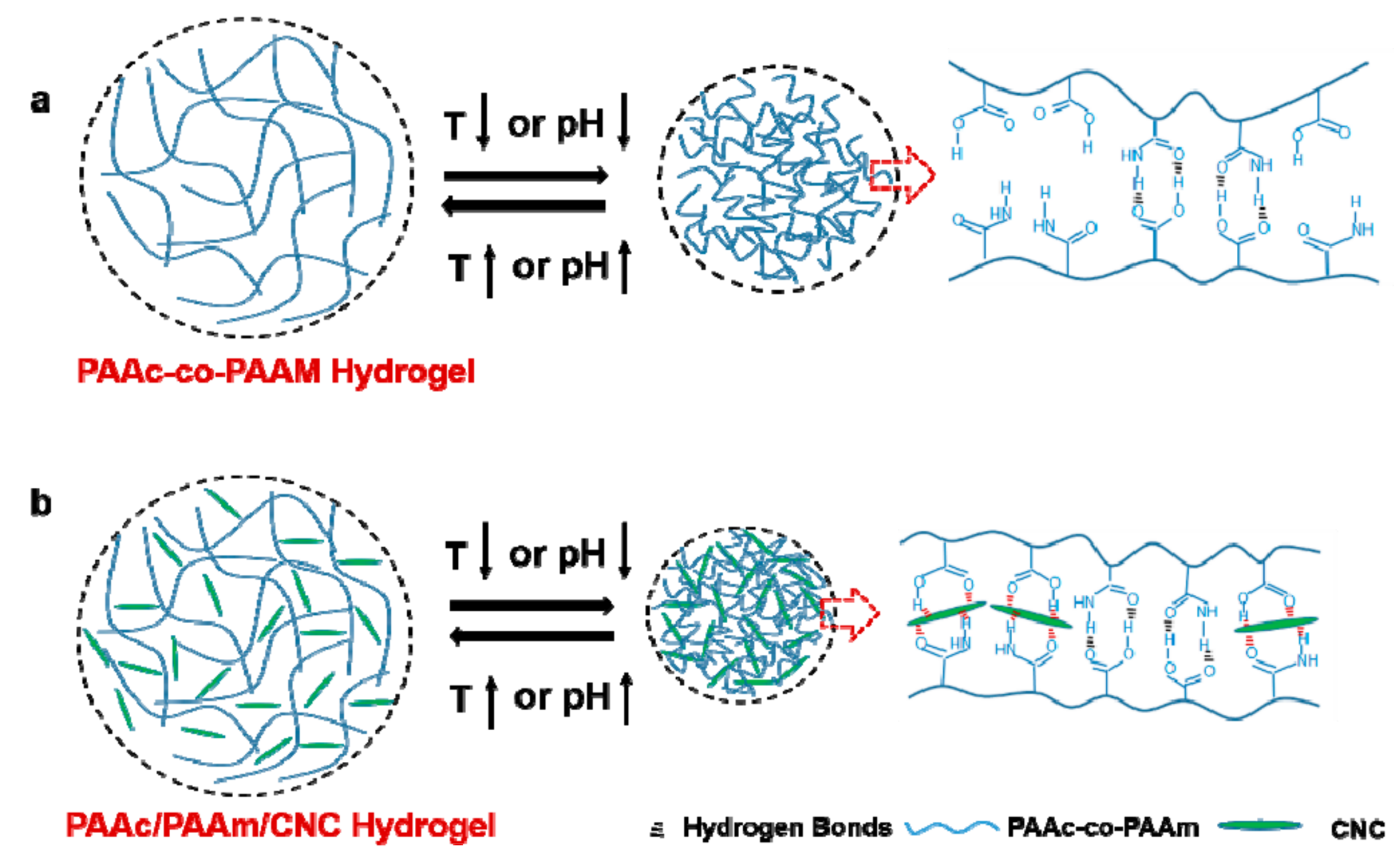
Schematic drawings of the microstructures in the PAAc-co-PAAm (**a**) and PAAc/PAAm/CNC hydrogels (**b**), indicating that the larger shrinkage of the latter, which is induced by the temperature/pH, is mainly due to the formation of hydrogen bonds between the polymer chains and CNCs.

As CNCs are the nanocrystalline form of cellulose, there are rich hydroxyl groups on their surface. When CNCs are incorporated and dispersed uniformly in the PAAc-co-PAAm matrix, many more H-bonding associations tend to form, such as the complex between –COOH and –CONH_2_ and the additional complexes between –OH and –COOH or –CONH_2_ during the cooling process (refer to Scheme 2). This prompts the polymer chains to be much more curled and compressed, greatly improving the shrinkage property of the hydrogel network after a decrease in the temperature. For the PAAc/PAAm/CNC_1_ sample, *R* reached 55.5%, while it was 58.4% for the PAAc/PAAm/CNC_2_ sample when both were reduced to an equilibrium temperature of 2 °C (Figure 3b). Figure 3c shows the structural representations of both the copolymer hydrogels without and with CNCs. These results indicate that as the temperature increases, the polymer–polymer or polymer–CNC complexes can be dissociated, and water enters due to the swollen hydrogel network; as the temperature decreases, these complexes can be reconstructed, and many water molecules are expelled.

During acid hydrolysis in the preparation of CNCs, sulfate ester groups (–OSO_3_H) are produced on the CNC surfaces. The sulfur content of the CNCs was evaluated to be 0.82 ± 0.04 wt% by a conductometric titration method [27]. According to the “Hofmeister series” [41,42,43], where –OSO_3_H is on the left of the Hofmeister series, CNCs are expected to tend to precipitate polymers through interactions with surface –OSO_3_H groups. To verify whether this was the case, the –OSO_3_H groups were removed by heating CNCs at 60 °C in 1 M NaOH for 5 h, and the resultant CNCs were named DCNCs. The sulphur content of the DCNCs was measured to be 0.27 ± 0.03 wt%. Instead of CNCs, an equal amount of DCNCs was used to prepare a PAAc/PAAm/DCNC_2_ hybrid hydrogel, where the corresponding components were identical to those in the PAAc/PAAm/CNC_2_ hydrogel. Then, the shrinkage behaviors of the two hydrogels were observed. Interestingly, from the photographs in Figure S3, the PAAc/PAAm/DCNC_2_ hydrogel demonstrated a weaker shrinkage ability than the PAAc/PAAm/CNC_2_ hydrogel at 2 °C, with a value of 30%. It is obvious that the –OSO_3_H groups play an important role in the shrinkage capability of the composite hydrogels. We speculate that the –OSO_3_H groups may promote the formation of complexes both between polymer and polymer and between the polymers and CNCs, which drives the hydrogels to shrink.

**Figure 4:**
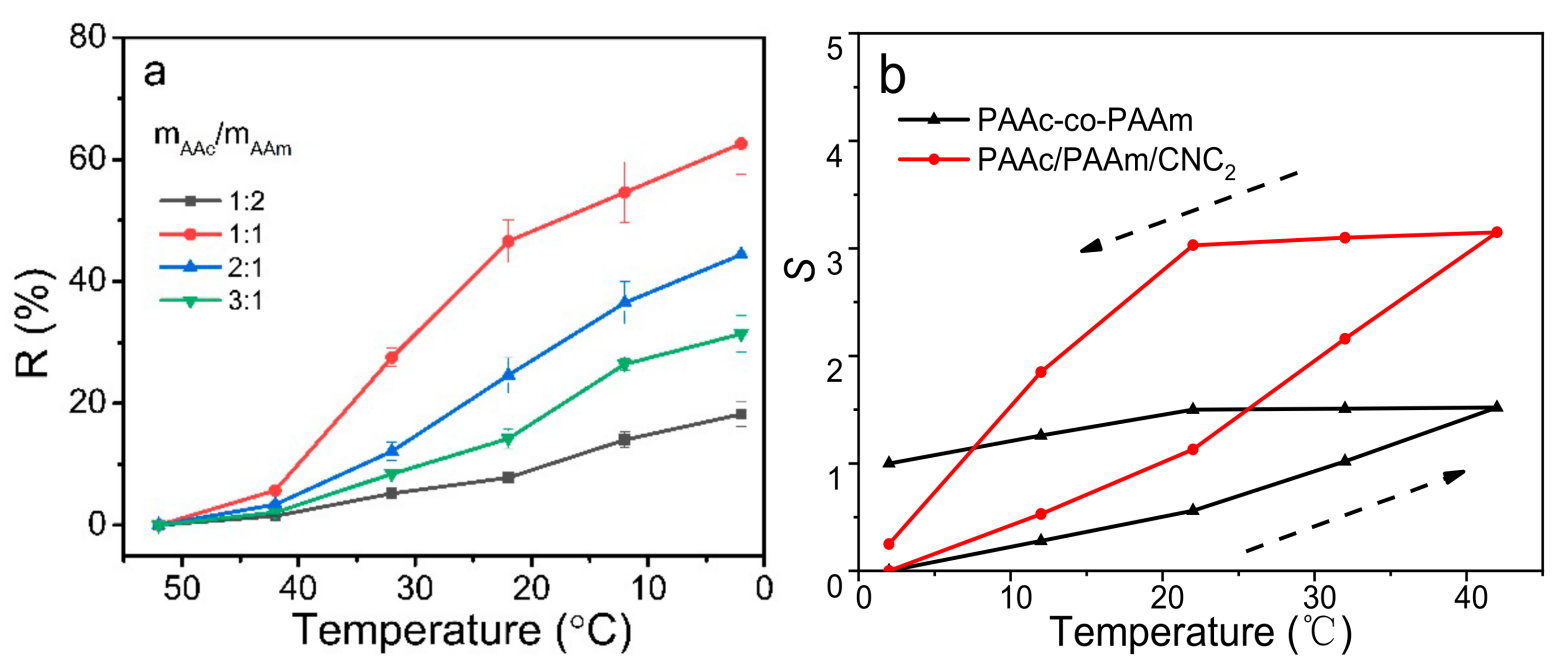
(**a**) Shrinkage ratio curves of the hydrogels at various mole ratios of AAc to AAm and varying temperatures, and (**b**) the temperature dependence of the equilibrium swelling ratio of the PAAc-co-PAAm and PAAc/PAAm/CNC_2_ hydrogels.

In addition to the amount of CNCs and their surface groups, the influence of the mole ratio of AAc to AAm (*n*_AAc_/*n*_AAm_) on hydrogel shrinkage was also considered. From Figure S4a, the PAAc/PAAm/CNC hydrogel with *n*_AAc_/*n*_AAm_ = 1 displayed a larger shrinkage capability than did those with *n*_AAc_/*n*_AAm_ values of 1/2, 1/3, and 2/1, and this result is consistent with the reports by Dai et al. and Ilmain et al. [30,37]. The shrinkage ratios corresponding to *n*_AAc_/*n*_AAm_ values of 1/2, 1/3, and 2/1 were approximately 18.1%, 31.4%, and 44.5%, respectively (Figure 4a). This result demonstrates that both the interactions between the PAAc and PAAm segments and those between the polymers and CNCs may contribute to the volume transition of hydrogels. In view of the optimal shrinkage capability, the PAAc/PAAm/CNC_2_ hydrogel with *n*_AAc_/*n*_AAm_ = 1 and 2 wt% CNCs was chosen as a typical sample for subsequent studies on volume transition and controlled release properties.

As discussed above, H-bonds are formed at low temperatures, while a high temperature causes the dissociation of polymer complexes in hydrogels, resulting from the breakage of H-bonds. This mechanism may further induce the inner network volume to expand and allow large amounts of water to enter the hydrogel phase from an ambient aqueous phase. Thus, a swelling ratio (*S*) was defined to evaluate the swelling property:

*S* = (*m_t_* − *m*_2_)/*m*_2_ (2)

where *m*_2_ is the weight of the hydrogel at 2 °C and *m_t_* is that at *t* °C. The *S* value of the PAAc/PAAm/CNC_2_ hydrogel gradually increased to 3.2 at 42 °C. From Figure 4b, one can see that when the temperature was reduced back to 2 °C, *S* was restored to 0.25, which is close to 0, indicating its good scalability and reversibility under temperature variation. Comparatively, the *S* value of the PAAc-co-PAAm hydrogel was merely 1.5 at 42 °C and restored to 1, reflecting its poor reversibility with low extensibility and retractility. Interestingly, from the images in Figure S4b, after a heating–cooling process, the PAAc-co-PAAm hydrogel was seriously crinkled, while the PAAc/PAAm/CNC_2_ hydrogel maintains its original 3D shape, i.e., a regular cylinder with a smooth surface, demonstrating the excellent free-standing property of the hydrogel when combined with CNCs.

**Figure 5:**
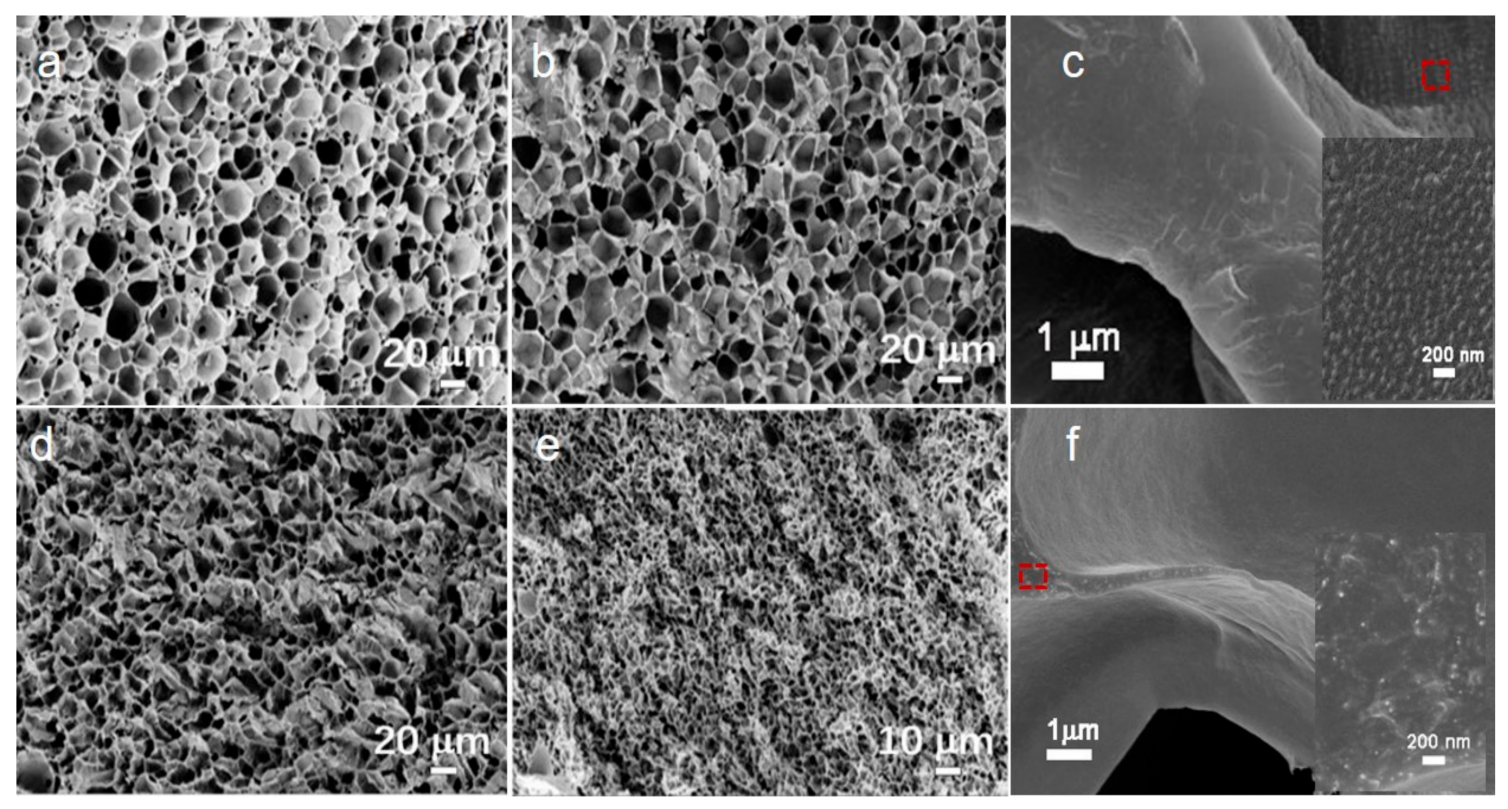
Cross-sectional SEM images. Hydrogels of PAAc-co-PAAm at (**a**) 42 °C and (**b**) 2 °C. Hydrogels of PAAc/PAAm/CNC_2_ at (**d**) 42 °C and (**e**) 2 °C. Morphologies of the pore wall of (**c**) the PAAc-co-PAAm and (**f**) PAAc/PAAm/CNC_2_ hydrogels at 2 °C; the inset images are magnified SEM images of the areas outlined by red frames.

The microstructures of the hybrid hydrogels at different temperatures were characterized by scanning electron microscopy (SEM), as shown in Figure 5. The interconnected porous morphology was viewed from the cross-sectional SEM images of all the hydrogels. For the PAAc-co-PAAm hydrogel, the average pore size only seemed to slightly decrease from 17.4 to 14.6 µm when the temperature changed from 42 °C to 2 °C (Figure 5a,b), which is consistent with the small shrinkage in hydrogel volume. As shown in Figure 5d,e, with the same temperature variation, the pore size of the PAAc/PAAm/CNC_2_ hydrogel was greatly reduced from 14.0 μm to 3.3 μm. Undoubtedly, the hydrogel network became tightly packed due to the large shrinkage effect. It was reported that the pore size of a hydrogel network is influenced by the cross-link density and the amount of water absorbed inside the hydrogel [8]. We may consider that the addition of CNCs provides multiple anchor sites for the PAAc-co-PAAm chains, which is responsible for the increase in cross-link density. As a result, less water can be held in the hydrogel. In addition, from the magnified SEM image, one can see that the pore wall and anchor sites of the PAAc-co-PAAm hydrogel (Figure 5c) are relatively smooth. In the case of the PAAc/PAAm/CNC_2_ hydrogel, many CNC nanorods are inserted into the pore wall and anchor sites and are arranged regularly, which makes the pore wall relatively rough (Figure 5f).

#### 3.2.2. pH Effects

**Figure 6:**
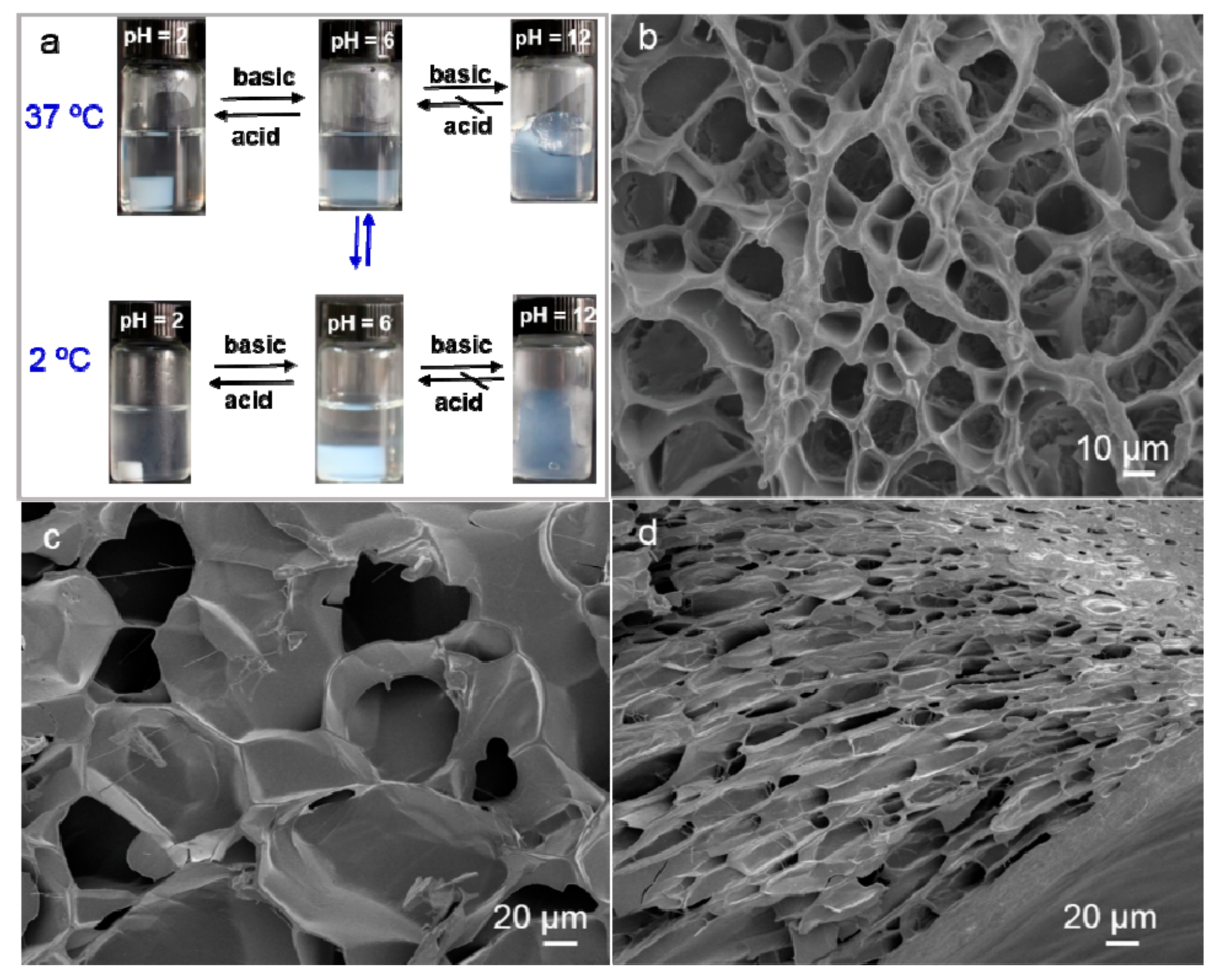
(**a**) Photographs of the swelling–shrinking behaviors of the PAAc/PAAm/CNC_2_ hydrogels at different pH values and at 37 and 2 °C. (**b**–**d**) Cross-sectional SEM images of the PAAc/PAAm/CNC_2_ hydrogels at varying pH values: pH = 2 (**b**), 6 (**c**), and 12 (**d**).

As mentioned above, theoretically, a significant portion of H-bonds are formed between the –COOH groups and–CONH_2_ groups. These bonds are important for the gelation and mechanical performance of hydrogels. The formation and dissociation of H-bonds, as well as the swelling and shrinking behaviors, can be tuned by altering the pH of the ambient solution. As shown in Figure 6a, at room temperature, a piece of hydrogel was immersed in water at different pH values, and reaching dynamic equilibrium usually took 4 h. As the pH changed from 2 to 6 and then to 12, the hydrogel expanded continuously, which is ascribed to the dissociation of both the PAAc–PAAm complex and H-bonds between polymers and CNCs. At high pH, –CONH_2_ and –COOH groups are deprotonated; thus, both polymer hydrophilicity and dissociation of the polymer complex are greatly enhanced [44,45]. As a result, the three-dimensional polymer network greatly expanded, and water molecules were absorbed into the hydrogel matrix. From the SEM image (Figure 6b,c), one can see that as the pH varied from 2 to 6, the pore size became significantly larger (from 18.3 to 122.7 µm), and the cross-linkage and pore density decreased correspondingly. At pH = 12, the rigidity of the hydrogel network was largely destroyed, with the pores seriously stretched and ruptured by excessive swelling (Figure 6d). It was determined that a pH above 8 causes permanent destruction of the PAAc/PAAm/CNC_2_ hydrogel, that is, the swollen hydrogel will no longer contract when it is replaced in a low-pH or low-temperature environment. For the swollen hydrogel at pH = 6 (<8), the volume decreased and the hydrogel became much more opaque when it was placed in water at a low pH or low temperature. Comparatively, in a low-pH environment (such as pH = 2), the PAAc/PAAm/CNC_2_ hydrogel exhibited reversible swelling–shrinking behavior upon switching of the temperature between 37 and 2 °C (Figure S5). Heating-induced swelling and cooling-induced shrinkage could be repeated at least 10 times without fatigue (Figure S5b).

### 3.3. Rheological Properties of PAAc/PAAm/CNC Hydrogels

**Figure 7:**
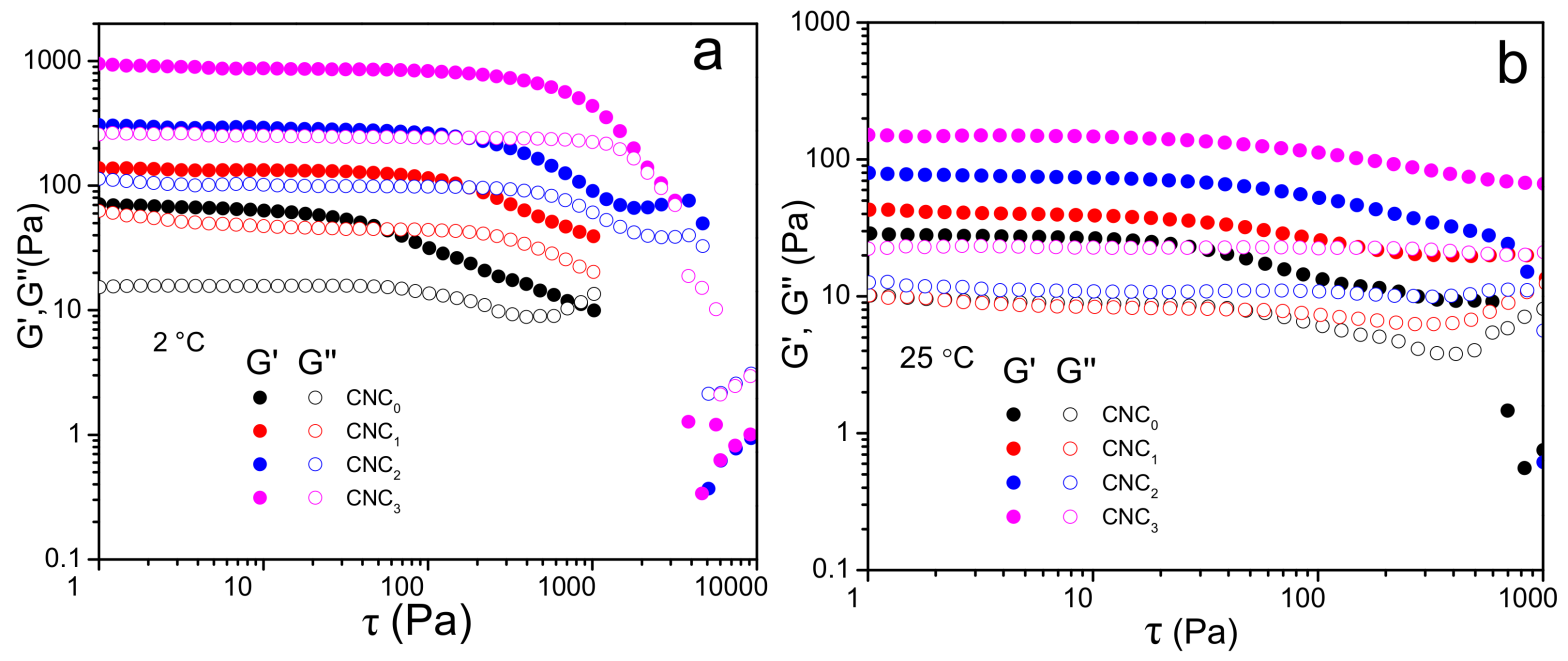
Stress sweep of the hydrogels with varying CNC concentrations at (**a**) 2 °C and (**b**) 25 °C.

The influence of CNC concentration and temperature on the mechanical strength of the PAAc/PAAm/CNC composite hydrogels was investigated, as shown in Figure 7. To determine the linear viscoelastic region of each hydrogel sample, the stress sweep was checked at a fixed frequency (1.0 Hz). As is well known, in the linear viscoelastic region, the elastic modulus (G′) is independent of the yield stress [46,47,48]. Additionally, the solid-like network structures of gels are suddenly broken when the yield stress is above a critical value (τ*). Typically, the critical value τ* reflects the strength of the network structures. Figure 7a illustrates that, at 2 °C, the τ* values of PAAc–PAAm, PAAc/PAAm/CNC_1_, PAAc/PAAm/CNC_2_, and PAAc/PAAm/CNC_3_ were about 50, 90, 200, and 500 Pa, respectively. This means that for hydrogels with a higher CNC concentration, the linear viscoelastic region became wider. With increasing CNC concentration, the elastic modulus (G′) also increased to reach a maximum value (1000 Pa) at 3% CNCs, which can be ascribed to more tightly entangled networks. At 25 °C (Figure 7b), the variation of the rheological properties of the composite hydrogels influenced by CNC concentration was similar to that at 2 °C. For pure PAAc–PAAm hydrogel, both the τ* and G′ values showed slight change with varying temperature, of about 20 and 30 Pa, respectively. For the PAAc/PAAm/CNC_3_ hydrogel, when the temperature changed from 2 to 25 °C, the τ* value showed nearly no change, while the G′ value decreased from 1000 to 200 Pa. One should note that for all hydrogels formed by PAAc/PAAm/CNC, the loss modulus (G″) was lower than G’ in the linear viscoelastic region; this elastic dominant behavior indicates typical reversible cross-linked networks in the hydrogels. As a general phenomenon, at the same temperature, with increasing CNC concentration, G″ increased to reach a highest value at 3% CNCs. 

### 3.4. Adsorption and Controlled Release of Dyes or Drugs

Stimulation-responsive intelligent hydrogels have been widely used to adsorb different types of toxic dyes from industrial wastewater [49,50,51,52,53]. In this work, two typical water-soluble dyes, negatively charged methyl orange (MO) and positively charged methylene blue (MB), were selected as simple models. Typically, 0.5 g of polymer hydrogel was immersed into 10 mL of dye solution at a concentration of 100 mg/L and then left undisturbed. Combined with the calibration curves of MB and MO (Figure S6a,b), the dye concentration after adsorption was monitored by UV–vis spectroscopy. The positively charged MB molecules were efficiently trapped in the hydrogels within two days, and the dark blue solution became clear, as shown in Figure S6c. The maximum uptake was measured to be 16.3 milligrams of MB per gram of hydrogel. However, for the negatively charged MO, the solution retained its dark-yellow color (Figure S6d), indicating the poor adsorption of MO in the hydrogel. Two reasons may explain the selective adsorption of MB. First, the surface of the composite hydrogel is negatively charged because of the sulfate ester groups on the CNCs, and strong electrostatic attraction can thus be formed between the hydrogel matrix and positively charged MB. Second, the pH of the MB solution was 7.5, and the hydrogel was expanded under this condition, which may have contributed to the adsorption of MB molecules.

It was reported that the solubility of polymers in water can be controlled by salts, which may influence the swelling–shrinking performance of the composite hydrogel, thus further controlling the release of adsorbed molecules. Aqueous solutions of two representative salts, NaCl and MgCl_2_, at different concentrations were used to trigger the shrinkage of the the PAAc/PAAm/CNC_2_ hydrogel at room temperature. From the photographs (Figure S7), the volumes of the PAAc/PAAm/CNC_2_ hydrogels were remarkably compressed by these salt solutions. First, large salt concentrations clearly led to high shrinkage ratios (*R*) for both the NaCl and MgCl_2_ solutions. Second, the triggered shrinkage ratios of the hydrogels gradually increased in the order of 0.2 mol/L NaCl, 0.1 mol/L MgCl_2_, 0.4 mol/L NaCl, and 0.2 mol/L MgCl_2_, which is exactly consistent with the increase in ionic strength.

**Figure 8:**
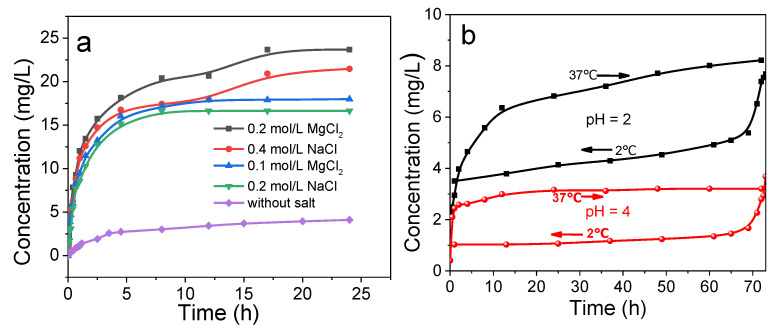
(**a**) Concentrations of methylene blue (MB) released from the as-prepared MB@PAAc/PAAm/CNC_2_ hydrogels in 10 mL of different salt solutions at room temperature as a function of time. (**b**) Release–resorption cycles of the MB@PAAc/PAAm/CNC_2_ hydrogel in 10 mL of water at two pH values and at 37 °C and 2 °C. The released concentration of MB was consecutively detected over time.

The release of MB from the as-prepared MB@PAAc/PAAm/CNC_2_ hydrogel (refer to Section 2.6) in pure water and different salt solutions was monitored over time. One can see from the release curves in Figure 8a that the initial release rate was comparatively rapid and then reached equilibrium after several hours. The addition of NaCl and MgCl_2_ enhanced the release of MB, since the equilibrium concentrations of MB were greatly improved. Furthermore, the released equilibrium concentrations also corresponded to the ionic strengths of the different salt solutions, implying that the release of MB can be simply tuned by the use of various salt solutions. The release of MB can also be tuned by changing the temperature and pH value. As shown in Figure 8b, at pH = 4, MB was released in ambient water at a concentration of ~3 mg/L at 37 °C. When the system was stored at 2 °C, MB was partially resorbed. At pH = 2, the release–resorption cycle at 37 °C and 2 °C was similar to that at pH = 4, but the equilibrium concentrations were much higher.

**Figure 9:**
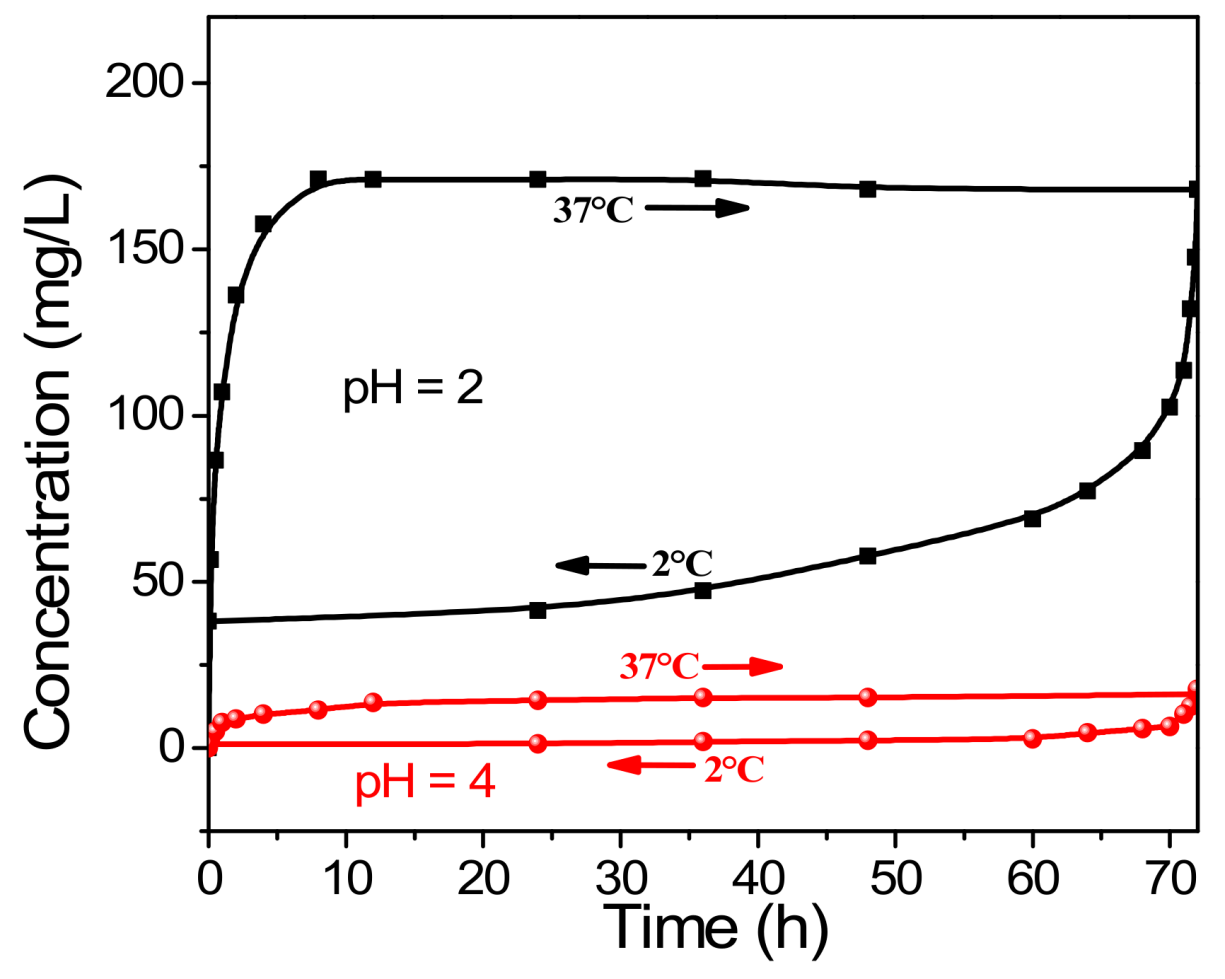
Release–resorption cycles of DOX-HCl from an as-prepared DOX-HCl@PAAc/PAAm/CNC_2_ hydrogel in 10 mL of water at pH = 2 or pH = 4 and at 37 °C and 2 °C. The released percentage of DOX-HCl was consecutively detected.

The above results inspired us to assess the controlled release of water-soluble ionic drugs through in vitro hydrogel shrinking and swelling tests. Doxorubicin hydrochloride (DOX-HCl), widely used as an anticancer drug [54], was selected in this work. An experiment to determine DOX-HCl release from the as-prepared DOX-HCl@PAAc/PAAm/CNC_2_ hydrogel in 10 mL of pure water was performed. The calibration curves of DOX-HCl are shown in Figure S8, and the release profile was obtained by detecting the time-dependent UV–vis spectra of the release media. As shown in Figure 9, the in vitro release studies with the PAAc/PAAm/CNC_2_ hydrogel revealed that the release behavior of DOX-HCl exhibited a trend similar to that of MB. At pH = 4, the released percentage of DOX-HCl at equilibrium in the aqueous phase was relatively low, i.e., 0.17% at 2 °C and 2.1% at 37 °C. Therefore, most of the DOX-HCl remained in the hydrogel phase, especially at a low temperature. When the pH value of the aqueous phase was 2, the released percentage of DOX-HCl in the aqueous phase increased, reaching 5.8% at 2 °C and 24% at 37 °C. Therefore, a high pH and low temperature are beneficial to the adsorption and storage of DOX-HCl, while in an acidic stomach environment (~37 °C and pH = 2), the DOX-HCl@PAAc/PAAm/CNC_2_ hydrogel can provide a high and stable release rate of DOX-HCl along with its gradual consumption. In addition, the release–adsorption behavior of the DOX-HCl@PAAc/PAAm/CNC_2_ hydrogel showed good reversibility: the cooling-induced adsorption and heating-induced release could be repeated at least 10 times without any decrease in the drug loading efficiency. The SEM image in Figure S9 shows that their structures were nearly unchanged. Based on the above results, the release–adsorption property makes this hydrogel a promising new candidate for targeted drug delivery and controlled release.

## 4. Conclusions

In conclusion, we successfully developed a multiresponsive UCST composite hydrogel based on PAAC-co-PAAM sequentially cross-linked with CNCs through hydrogen bonds. The microstructures of the PAAc-co-PAAm/CNC hybrid hydrogels were densely cross-linked porous structures. The pore size varied with the volume transition of the hydrogels. With tuning of the environmental temperatures, pH values, and salt concentrations, the PAAc-co-PAAm/CNC hybrid hydrogels exhibited swelling and shrinking. Particularly, at pH = 2, cooling-induced shrinking and heating-induced swelling could be repeated at least 10 times without damaging the structure and properties of the hydrogel. Compared to the PAAc-co-PAAm hydrogel, the shrinkage performance and toughness of the PAAc/PAAm/CNC hydrogels were significantly improved. These excellent properties of the composite hydrogel inspired us to utilize the shrinkable polymer hydrogel as an intelligent carrier for the efficient adsorption of ionic dyes and the controlled release of drugs. A high pH and low temperature were beneficial to the adsorption and storage of drugs in the PAAc/PAAm/CNC hydrogels, while a low pH and high temperature were helpful for the release of drugs from the hydrogels. These hybrid hydrogels are expected to be promising candidates for drug delivery and controlled release.

## Data Availability

The data presented in this study are available on request from the corresponding author.

## Supplementary Materials

The following are available online at https://www.mdpi.com/article/10.3390/polym13081219/s1, Figure S1: Photographs of the typical hydrogels at 52 °C. From left to right: hydrogels of PAAc-co-PAAm, PAAc/PAAm/CNC_1_, PAAc/PAAm/CNC_2_, and PAAc/PAAm/CNC_3_. Figure S2: DSC curves of CNCs and the PAAc-co-PAAm and PAAc/PAAm/CNC_2_ hydrogels in the whole temperature range. Figure S3: Photographs of the PAAc-co-PAAm/DCNC_2_ hydrogels at 52 °C and 2 °C. Figure S4: Photographs of (a) PAAc-co-PAAm/CNC hydrogels with varying mole ratios of AAc to AAm at a fixed CNC concentration of 2%, and (b) PAAc-co-PAAm and PAAc/PAAm/CNC_2_ in the initial state and after a heating–cooling process in the final state. Figure S5: (a) Reversibility of the PAAc/PAAm/CNC_2_ hydrogel after 10 cycles at 37 and 2 °C; (b) SEM image of PAAc/PAAm/CNC_2_ hydrogels after 10 cycles of cooling and heating. Figure S6: Calibration curves of (a) MB and (b) MO in aqueous solutions, and the MB (c) and MO (d) solutions before (left) and after (right) adsorption. Figure S7: (a) Photographs of PAAc/PAAm/CNC_2_ hydrogels in water (top) and in different salt solutions (bottom). (b) Shrinkage ratios of PAAc/PAAm/CNC_2_ hydrogels in different salt solutions to those in pure water. All systems were equilibrated at room temperature for 2 days. Figure S8: Calibration curve of DOX-HCl in aqueous solution. Figure S9. Cross-sectional SEM image of the dried DOX-HCl@PAAc/PAAm/CNC_2_ hydrogel after 10 cycles of cooling-induced adsorption and heating-induced release by alternating the temperature between 2 °C and 37 °C.
