# Supplementary material for: Multiresponsive Cellulose Nanocrystal Cross-Linked Copolymer Hydrogels for the Controlled Release of Dyes and Drugs
Source: Polymers (Basel). 2021 Apr 9;13(8):1219. doi: 10.3390/polym13081219 (PMC8070268; doi:10.3390/polym13081219)
Supplement: Supplementary file 1 [file polymers-13-01219-s001.pdf]

Supplementary material

Multiresponsive Cellulose Nanocrystal Cross-Linked Copolymer Hydrogels for the Controlled Release of Dyes and Drugs

Yuchen Jiang,[1] Guihua Li,[1] Chenyu Yang,[1]Fangong Kong and Zaiwu Yuan[1,2*]*

[1] Key Laboratory of Fine Chemicals in Universities of Shandong, School of Chemistry and Chemical Engineering, Qilu University of Technology (Shandong Academy of Sciences), Jinan 250353, China

[2] State Key Laboratory of Biobased Material and Green Papermaking, Qilu University of Technology (Shandong Academy of Sciences), Jinan 250353, China

[*]To whom correspondence should be addressed.

E-mail: yuanzaiwu@163.com, Z. Yuan

liguihua19880922@163.com, G. Li

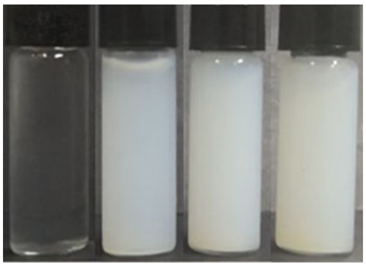

Fig. S1. Photographs of the typical hydrogels at 52 °C. From left to right: hydrogels of PAAc-co-PAAm, PAAc/PAAm/CNC_1, PAAc/PAAm/CNC_2, and PAAc/PAAm/CNC_3.

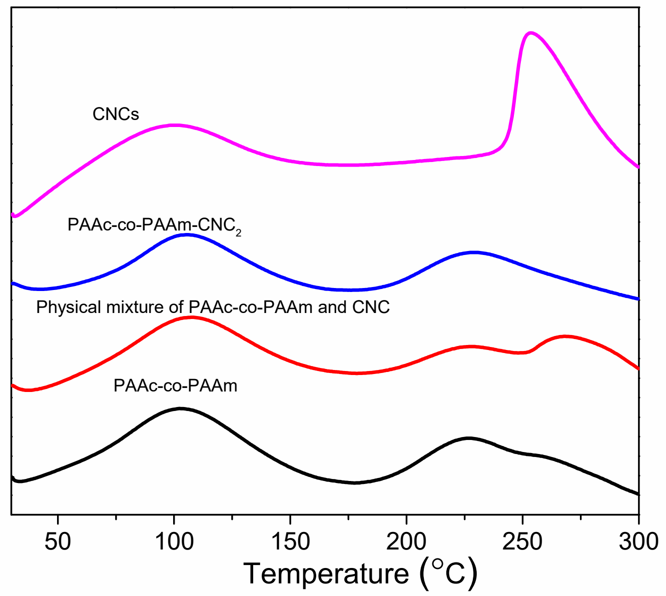

Figure S2. DSC curves of CNCs and the PAAc-co-PAAm and PAAc/PAAm/CNC_2 hydrogels in the whole temperature range.

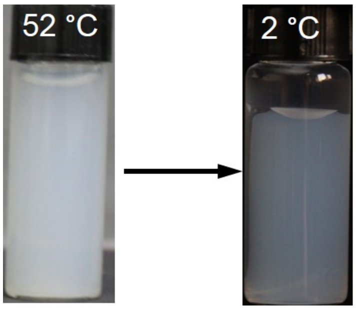

Fig. S3. Photographs of the PAAc-co-PAAm-$DCNC_2$ hydrogels at 52 °C and 2 °C.

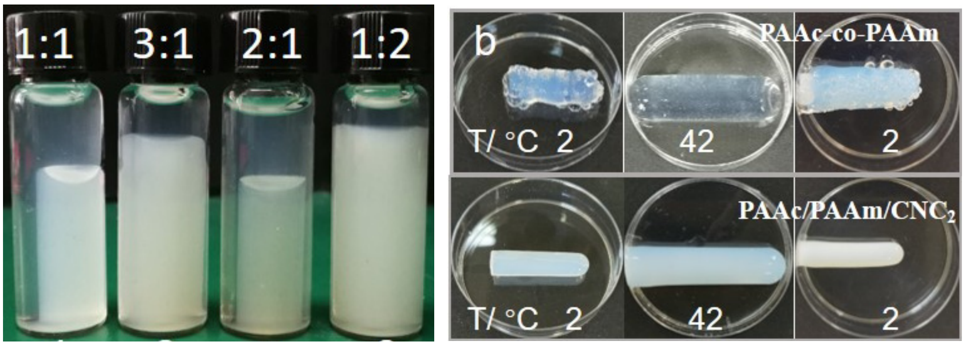

Fig. S4. Photographs of (a) PAAc-co-PAAm/CNC hydrogels with varying mole ratios of AAc to AAm at a fixed CNC concentration of 2%, and (b) PAAc-co-PAAm and PAAc/PAAm/CNC2 in the initial state and after a heating–cooling process in the final state.

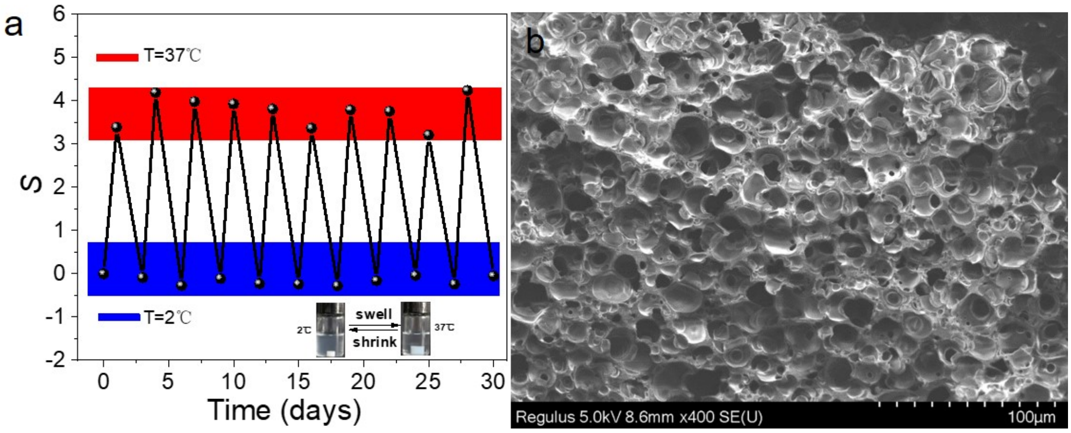

Fig. S5. (a) Reversibility of the PAAc/PAAm/CNC2 hydrogel after 10 cycles at 37 and 2 °C; (b) SEM image of PAAc/PAAm/CNC2 hydrogels after 10 cycles of cooling and heating.

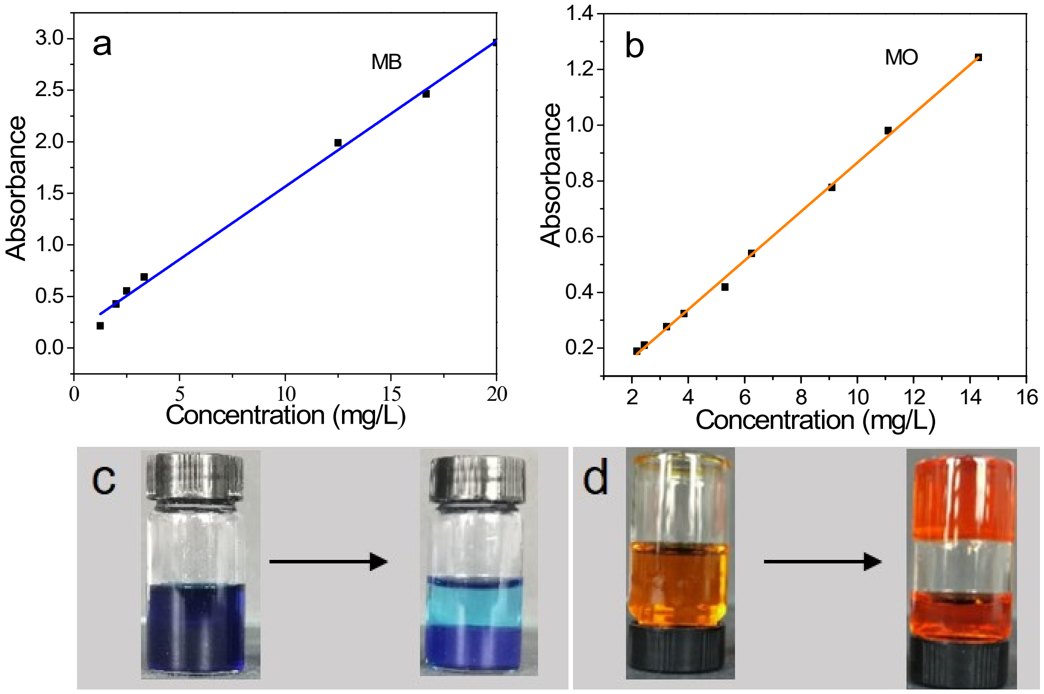

Fig. S6. Calibration curves of (a) MB and (b) MO in aqueous solutions, and the MB (c) and MO (d) solutions before (left) and after (right) adsorption.

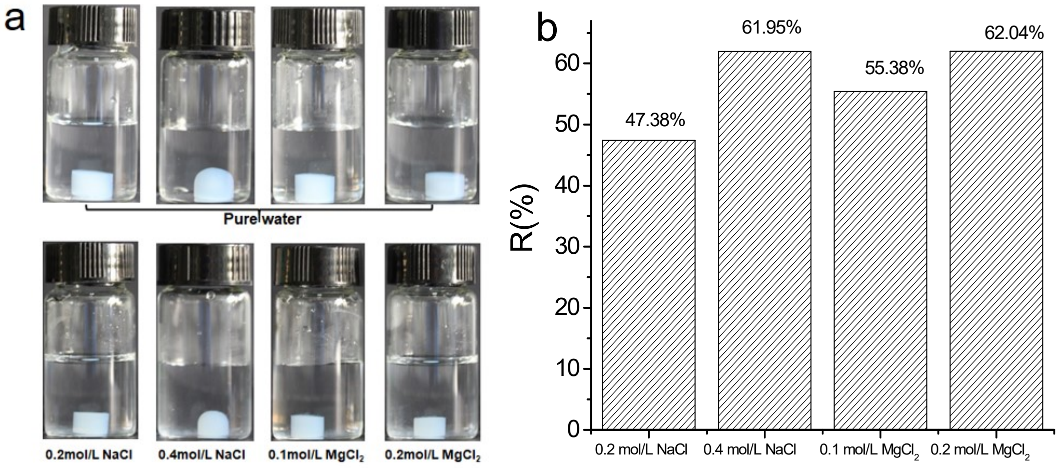

Fig. S7. (a) Photographs of PAAc/PAAm/CNC_2 hydrogels in water (top) and in different salt solutions (bottom). (b) Shrinkage ratios of PAAc/PAAm/CNC_2 hydrogels in different salt solutions to those in pure water. All systems were equilibrated at room temperature for 2 days.

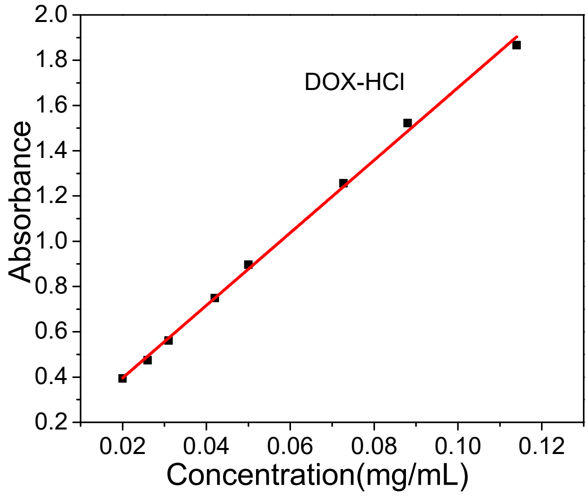

Fig. S8. Calibration curve of DOX-HCl in aqueous solution.

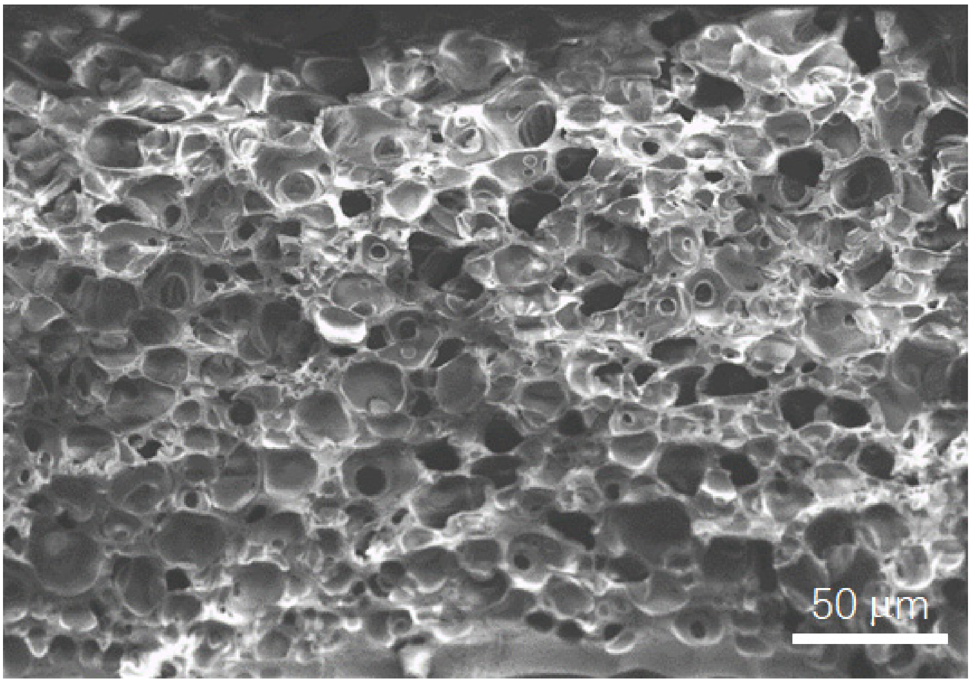

Fig. S9. Cross-sectional SEM image of the dried DOX-HCl@PAAc/PAAm/CNC_2 hydrogel after 10 cycles of cooling-induced adsorption and heating-induced release by alternating the temperature between 2 °C and 37 °C.
